# A Mixed-Methods Realist Evaluation of the Implementation and Impact of Community Forensic CAMHS to Manage Risk for Young People With Forensic and Mental Health Needs: Study Protocol

**DOI:** 10.3389/fpsyt.2021.697041

**Published:** 2021-11-04

**Authors:** Rebecca Lane, Sophie D'Souza, Maria Livanou, Jenna Jacob, Wendy Riches, Roz Ullman, Anisatu Rashid, Rosie Singleton, James Wheeler, Peter Fuggle, Dickon Bevington, Jessica Deighton, Duncan Law, Peter Fonagy, Nick Hindley, Oliver White, Julian Edbrooke-Childs

**Affiliations:** ^1^Anna Freud National Centre for Children and Families, Kantor Centre for Excellence, London, United Kingdom; ^2^Clinical, Educational, and Health Psychology, University College London, London, United Kingdom; ^3^Child Outcomes Research Consortium, Evidence Based Practice Unit, Kantor Centre for Excellence, University College London and the Anna Freud National Centre for Children and Families, London, United Kingdom; ^4^Department of Psychology, School of Law, Social and Behavioural Sciences, Kingston University, London, United Kingdom; ^5^Riches and Ullman Limited Liability Partnership (LPP), Wallington, United Kingdom; ^6^MindMonkey Associates Ltd., London, United Kingdom; ^7^South Central Forensic Child and Adolescent Mental Health Services (F:CAMHS), Oxford Health National Health Service (NHS) Foundation Trust, Oxford, United Kingdom; ^8^Former National Community Forensic Child and Adolescent Mental Health Services (F:CAMHS) Clinical Lead, National Health Service (NHS) England and NHS Improvement, London, United Kingdom; ^9^Southern Health National Health Service (NHS) Foundation Trust, Calmore, United Kingdom; ^10^South West (North) Community Forensic Child and Adolescent Mental Health Services (F:CAMHS), Oxford Health National Health Service (NHS) Foundation Trust, Keynsham, United Kingdom; ^11^National Community Forensic Child and Adolescent Mental Health Services (F:CAMHS) Clinical Lead, NHS England and NHS Improvement, London, United Kingdom

**Keywords:** mental health, forensic psychiatry, child and adolescence, protocol, realist evaluation

## Abstract

**Introduction:** Young people in contact with forensic child and adolescent mental health services present with more complex needs than young people in the general population. Recent policy has led to the implementation of new workstreams and programmes to improve service provision for this cohort. This paper aims to present the protocol for a national study examining the impact and implementation of Community Forensic Child and Adolescent Mental Health Services (F:CAMHS).

**Methods and analysis:** The study will use a mixed-methods Realist Evaluation design. Quantitative service activity and feedback data will be collected from all 13 sites, as well as questionnaires from staff. Non-participant observations and qualitative interviews will be conducted with staff, young people and parents/guardians from four focus study sites. An economic evaluation will examine whether Community F:CAMHS provides good value for money. The results will be triangulated to gain an in-depth understanding of young people's, parents/guardians' and staff experiences of the service.

**Ethics and dissemination:** Ethical approval was granted by the Health Research Association and UCL Ethics. The results will be disseminated via project reports, feedback to sites, peer-reviewed journal publications and conference presentations.

## Introduction

Research suggests that young people experiencing multiple and sustained risk factors are at greater likelihood of experiencing mental health difficulties and deleterious outcomes (1). Research has found that young people who present with high risk of harm to self and/or others have high levels of psychosocial adversity, neurodevelopmental disorders and learning difficulties, multiple mental health difficulties and co-morbid needs, and substance misuse (2, 3). Correspondingly, they may be in contact with mental health, social, or specialist support services and experience frequent transitions between services and geographical displacement. These factors combined likely make this cohort particularly vulnerable to exploitation. Furthermore, there may be particular barriers to service engagement for some children and young people, including those in contact with the criminal justice system (4), those for whom English is not their first language (5), or those from minoritized ethnic backgrounds (5, 6), who are over-represented in youth justice services (7). Systemic and structural issues affecting service engagement have also been identified. These include logistical barriers leading to children being underserved by services; e.g., location of service, long wait times and lack of specialist services (8). Indeed, previous research has highlighted that children and young people who present with high risk of harm to others or to self have commonly not been in contact with child and adolescent mental health services (CAMHS), despite being known to have high rates of mental health difficulties (9). Such social disadvantage and lack of contact with support place young people who present with high risk of harm to self and/or others at further risk of behaviours that might result in contact with criminal justice systems, which can have a considerable negative impact on life chances (1).

Recent policy in child and adolescent mental health, for instance, Future in Mind and the 5 year Forward View (10, 11), highlight the need for more research into young people with high risk of harm to self and/or others and for services to manage risk. Research suggests that youth services, such as outpatient CAMHS, do not have the capacity or scope to identify, assess, and address the multiple and very complex needs of this population (12). Particular mental healthcare needs may be difficult to meet through general and existing specialist services due to the unique and complex circumstances of this population (11).

Further, the provision of services for young people who present with high risk of harm to self and/or others across agencies across England has been argued to be fragmented and lacking in coordination (13, 14), leaving large areas of England with no access to any specifically commissioned forensic mental health service for children and young people (13). To address this need, as part of the Health and Justice and Specialised Commissioning Children and Young People Mental Health Transformation Workstream (15), 13 new Community Forensic Child and Adolescent Mental Health Services (F:CAMHS) have been commissioned by NHS England and NHS Improvement. The aim is to address gaps in support, to provide consistent Community F:CAMHS provision nationally, and to divert young people from the youth justice system. Community F:CAMHS are small teams providing highly specialist input to the network around children and young people who present with a high risk of harm to self and/or others, or who are in contact with the youth justice system, about whom there are questions or concerns regarding mental health and/or a learning disability (14). Broadly, Community F:CAMHS provide three types of support: advice and consultation, when professionals are concerned about a young person; case co-ordination for particular young people involved with a number of different organisations; and direct clinical work for a small number of young people with complex cases and who need highly specialised assessment and intervention (14).

Two evaluations of existing early, small scale Community F:CAMHS in the Thames Valley/Hampshire and Isle of White have already been conducted (9, 16). These evaluations analysed service activity data and collected feedback from professionals in contact, or likely to be in contact, with the service, to assess levels of satisfaction and to highlight possible areas of improvement. Professionals who referred into the services were able to identify cases where the involvement and support of Community F:CAMHS likely resulted in savings for NHS commissioners, for example due to reduced hospital stays or out of area placements (16). Furthermore, interviewees reported that Community F:CAMHS addressed gaps in service provision and decreased the potential for vulnerable young people to fall through these gaps. The findings concluded that Community F:CAMHS have an important role to improve clinical governance and risk assessment, provide strategic advice, and to support young people and their network with the transitions between services. Further research is required to explore the impact of Community F:CAMHS on young people and their parents/guardians.

The new Community F:CAMHS are modelled on the earlier, regional services (14, 16). However, there is a gap in the literature in relation to how this provision will work nationally and in different local contexts. There is also a literature gap in exploring the impact of Community F:CAMHS on young people's outcomes and experience of accessing these services.

### Aims

The aims of the present study are to address the above research gaps and to examine whether the implementation of the new national service specification for high risk young people leads to an improved understanding of need and improved case coordination and support. The primary research questions are:

1. What Are the Characteristics of Young People Accessing Community F:CAMHS?
2. What Are the Outcomes and Experiences of Young People Accessing Community F:CAMHS?
3. What Are the Experiences of Staff (Including Professionals in Contact With the Service) Working in Community F:CAMHS?
4. What Is the Cost Effectiveness of Community F:CAMHS?

## Methods and Analysis

### Study Design and Recruitment

The overall approach will be a 3-year longitudinal prospective Realist Process Evaluation (17) using a mixed-methods design. All sites will fully implement the new Community F:CAMHS, via a process of mobilisation, recruitment, and service delivery. All sites have been commissioned to implement Community F:CAMHS fully, however some sites were able to recruit staff, engage stakeholders, and deliver their service at an earlier point than others. Therefore, some sites are termed “early implementers” while others are “late implementers”.

Quantitative data will be collected in all 13 services. Qualitative data collection will take place at four focus study sites (two early implementers, two late implementers) identified based on their progress with implementation (e.g., recruitment, service activity), service maturity, and geographical spread to explore experiences of implementation and of service. Non-participant observations will be completed to examine the extent to which services are implementing Community F:CAMHS as expected and whether changes are sustained over time. An economic evaluation will take place to examine whether Community F:CAMHS provides good value for money (see “Economic analysis” section below).

The quantitative strand using questionnaires, feedback data, and routine service data will be used to examine the characteristics, outcomes, and experiences of young people accessing these services, and the experiences of staff implementing Community F:CAMHS. All young people referred to Community F:CAMHS will be included in the routine service data. Community F:CAMHS staff (e.g., nurses, psychiatrists, psychologists, social workers, occupational therapists, administrators) will be eligible to participate in the staff questionnaires. Staff working with these settings (including referring and receiving services) will be approached to provide feedback on their experience.

The qualitative strand will be used to examine services' journeys through the stages of implementation (i.e., exploration and adoption, program installation, initial implementation, full operation, innovation and sustainability) (18) and young people's views on the impact of accessing Community F:CAMHS and their experiences. Children and young people aged 16 and above with direct contact with Community F:CAMHS, and their parents/guardians, will be eligible to participate in the interviews or focus groups. Community F:CAMHS staff and staff working with these services (including referrers) will be eligible to participate. Children and young people who are not able to provide informed consent (i.e., Gillick competent) will not be eligible to take part in the interviews.

### Sample Size

With 5% significance and 80% power, we will need to collect data on 26 young people in the early implementer sites and 26 young people in the late implementer sites to be able to detect a clinically meaningful difference in mental health using our primary outcome measure, the Health of the Nation Outcome Scales for Children and Adolescents (HoNOSCA) (19). Young people's scores in the late implementer sites have an estimated score of 15.51 and in the early implementer sites have an estimated score of 11.18 (note: higher scores indicate worse mental health) (19). As the data are clustered within services and we estimate the intraclass correlation coefficient to be 20%. We would need to recruit a minimum of 6 services and 120 young people in the early implementer sites and 6 services and 120 young people in the late implementer sites (i.e., 12 services and 240 young people).

For qualitative data, we anticipate recruiting in each focus study site 7–10 staff members at three time-points and 10–15 young people or parents/guardians per focus site over the course of the Realist Evaluation. The sample size was determined based on the research team's rich experience of conducting similar interviews to achieve data saturation.

### Patient and Public Involvement

The study will use Patient and Public Involvement throughout the study. Professionals, parents and young people will be consulted in focus groups to review study materials, for example participant information sheets, and survey materials to ensure the accessibility of the materials and to maximise value of the data collected. The Expert Panel and Steering Groups, which include young people with lived experiences and expert clinicians, will aid clinical interpretations of the data. Young people and Local Collaborators will be involved in dissemination of findings through events in their sites at all stages of the study.

## Data and Analysis

### Implementation Data

#### Implementation Description

**Figure 1:**
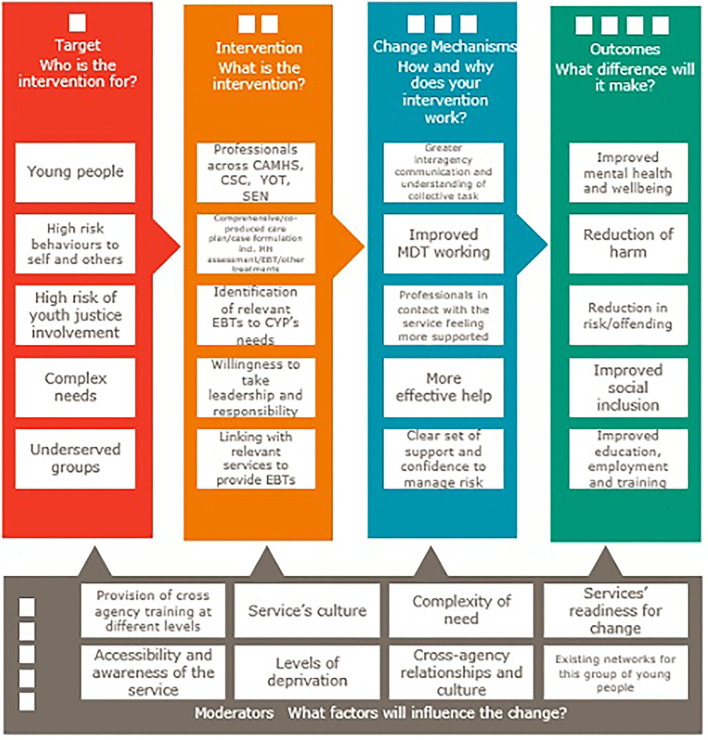
Logic model for community F:CAMHS evaluation.

As a guide to provide a rich, comprehensive description of the implementation of Community F:CAMHS, staff will be supported with completion of the Template for Intervention Description and Replication (TIDieR) (20) framework, a widely used tool to describe interventions in sufficient detail to allow their replication. A logic model is used to model expected change in outcomes as a result of the intervention of F:CAMHS and identify any moderators which may influence change (see Figure 1). Through the logic model and TIDieR framework, we will achieve a detailed understanding of the Core Components, crucial within implementation science (18).

#### Observations

The research team will conduct non-participant observations at early, mid and late evaluation stages using an observational tool. The tool has been developed specifically to capture the team processes that take place during team meetings and comprises four domains, each of which is rated on a five-point scale, from 1 (strongly disagree) to 5 (strongly agree), with free text response sections for notes: Structure (i.e., “The MDT meeting followed a clear structure”), Integration of Service Provision (i.e., “The meeting was focused on integrating service provision to meet the young person's needs”), Collaborative Culture (i.e., “Everyone had the opportunity to contribute and all points of view were respected”), and Risk Management (i.e., “There were opportunities to identify risks and discuss concrete plans to mitigate these risks”).

### Quantitative Data

Two strands of quantitative data will be collected at all 13 sites: routine service data and staff questionnaires.

#### Routine Service Data

Routine service data collected by sites will be anonymized and shared in line with data sharing agreements between the sites and the research team. It will involve data such as: referral source, presenting difficulties, risk factors (e.g., fire setting, adverse childhood experiences, trauma), contact type with young people (e.g., indirect contact vs. direct contact), contact duration, type of professionals involved, service types involved, integrated care plan in place, case closure reason, young person's care setting and placement stability and the service's feedback received from referrers, young people and parent/guardians.

The routine data will also include clinical data drawn on the following constructs:

**Mental health and wellbeing**. Our primary measure of mental health and well-being is the HoNOSCA (19). The HoNOSCA is a clinician-rated, 13-item questionnaire which measures change in severity of difficulties over time in accordance with retrospective clinical judgement. The items are split into subgroups of Behavioural Problems, Impairment, Symptomatic Problems, and Social Problems and are rated on a scale of 0–4 (“No problem” to “Severe to very severe problem”). The HoNOSCA has good inter-rater reliability and shows satisfactory coverage, internal structure and its total score relates well to case severity (21). Upon consultation with the evaluation Steering Group and participating sites, the HoNOSCA was deemed appropriate as it covers a range of psychosocial or behavioural difficulties which children and young people in contact with Community F:CAMHS may present with. Using the HoNOSCA will avoid the use of multiple scales to capture the broad range of difficulties that children and young people presenting with high risk, high harm, high vulnerability may experience, which supported our efforts to not overburden sites and encourage site engagement. Our second measure of well-being is the Child Outcome Rating Scale (CORS) (22), a four-item self-rated visual analogue scale that assesses symptom distress, interpersonal relationships, functioning, and global well-being. It is widely used in youth mental health clinical work, research, has established clinical cut offs and has demonstrated reliability and validity (23).

**Quality of life/overall health**. This will be measured using the *EQ-5D-Y* (24) proxy version, which is a measure of health status and quality of life. The descriptive system comprises the 5 child-friendly dimensions of quality of life (mobility, looking after myself, doing usual activities, having pain or discomfort, and feeling worried, sad or unhappy) which are rated on a three-point scale: “no problems,” “some problems,” “a lot of problems.” The visual analogue scale records overall health, where the endpoints are labelled “The best health you can imagine” and “The worst health you can imagine.” Research has demonstrated good correlation and convergent validity of the EQ-5D-Y.

**Experience of service**. The self-report *Experience of Service Questionnaire* (ESQ) (25) will be used to assess children and young people's satisfaction with care and satisfaction with the environment (26). Nine of the original 12 items (statements 1, 2, 3, 4, 5, 6, 7, 9 and 10) appropriate to a community consultation service will be used in the current study, integrated into a service feedback form. Responders are asked to rate their agreement with statements on a 4-point scale of either “Certainly true,” “Partly true,” “Not true” or “Don't know.” Four of the included items will be used as a proxy for measuring shared decision-making, as administered in previous research (27). The ESQ is widely used in CAMHS settings in the UK, and has demonstrated inter-rater reliability and construct validity (26). Given its routine use in services of this nature, it is an appropriate and realistic measure to use. Using a familiar measure is more likely to reach saturation than the introduction of a new and less widely-used and recognised measure in this context. This measure has also been used in other similar studies to assess the experience of children and young people accessing mental health services [e.g., (27)].

#### Staff Questionnaires

Questionnaires will be collected from staff at three time-points during the study. The questionnaires will include demographic information, as well as bespoke items about staff views of Community F:CAMHS, training and supervision. They will also include information on the following constructs:

**Staff burnout**. Staff will be asked to complete the *Copenhagen Burnout Inventory* (CBI) (28), a 19-item tool consisting of three scales: personal burnout, work-related burnout, and client-related burnout. This will also cover areas such as resilience and coping. Respondents are asked to score items on a 5-point scale ranging from “Always,” to “Never/almost never” or from “To a very high degree,” to “To a very low degree.” All three scales of the CBI have been found to have very high internal reliability, and low non-response rates (28).

**Self-efficacy**. The *Risk Assessment and Management Self-Efficacy Scale* (RAMSES) (29) will be used to measure staff confidence and self-efficacy. The RAMSES is specifically designed for risk management in mental healthcare, following Bandura's theory of developing measures of self-efficacy (30). The RAMSES contains a total of 18 items subdivided into three broad domains: assessment, management and referral. Respondents are asked to rate their perceived self-efficacy on a Likert scale ranging from 0 (no confidence in ability) to 10 (complete confidence in ability). Evidence of adequate internal consistency, construct and discriminant validity have been described for this measure (29).

**Staff satisfaction**. Items on staff satisfaction and experience will be drawn from the national NHS Staff survey 2017, an independent survey of employees' experience of working. It captures background information, work attitudes, their managers, information on their health, well-being and safety at work, their personal development and their organisation.

**Service climate**. To measure service climate and team function, we will be using seven items from the revised *Team Climate Inventory* (TCI) (31) that measure Participatory Safety and Support for Innovation. Respondents are asked to score each item on a 5-point scale ranging from “Strongly disagree” to “Strongly agree.” Research has supported the internal homogeneity, reliability and normality of the scales, and suggests comparative predictive validity between the shortened TCI and the original version (32). To measure social and therapeutic climate, we will be using 5 items from the EssenCES (33) that measure Therapeutic Hold. Respondents are asked to score each item on a 5-point scale ranging from “Not at all” to “Very much.” Research has found satisfactory internal consistency for all EssenCES scales and supports its construct, convergent and divergent validity (34).

#### Analysis

Descriptive statistics will be conducted using the anonymized routine service data and responses from the questionnaires. We will seek to explore the complexities and clinical presentations of this population, compared, where appropriate and fitting, to the wider CAMHS population, using published comparator information. The primary analysis will be a multilevel regression predicting change in mental health using the HoNOSCA with early vs. late implementation as a predictor variable, accounting for the clustered structure of the data (with time clustered within young people clustered within services) and controlling for covariates (e.g., age, gender, ethnicity, number of risk factors). We will additionally explore change over time by conducting *t*-tests and ANOVAs using the anonymized routine service data and responses from the questionnaires.

### Qualitative Data

Qualitative data will be collected from staff and young people or parents/guardians at the four focus study sites.

#### Staff

Semi-structured interviews/focus groups will be conducted with staff at three-time points. The topic guide will explore services' journey through the stages of implementation and the levels at which culture change is occurring, from expressed or reported, lived and experienced and deep or ingrained in the organisational structure. Interviews will also explore the mechanisms and barriers/facilitators to implementation and staff experience, including coping and resilience. The topic guide will also explore experiences and the impact of recent global events, i.e., the coronavirus pandemic and the Black Lives Matter protests.

#### Young People and Parents/Guardians

Semi-structured interviews will be conducted with young people and families (in case the young person lacks mental capacity and/or has a severe learning disability) throughout the study. The topic guide will explore young people's perspective on the levels at which cultural change is occurring, views and experiences of the services and implementation mechanisms (e.g., improved assessment of needs, better transitions and better experience of care). The topic guide will also explore experiences and the impact of recent global events, i.e., the coronavirus pandemic and the Black Lives Matter protests.

##### Analysis

Interviews/focus groups will be audio-recorded and transcribed verbatim. Transcripts will be analysed using framework analysis (35) and thematic analysis (36) and coded using NVivo software according to established methods for qualitative analysis validation (37).

Mixed-methods matrix allows researchers to collect quantitative and qualitative data that are integrated during the analysis stage, with data from different sources being studied together. Within a mixed methods matrix, the rows display the cases for which there is both qualitative and quantitative data, and the columns demonstrate different data collected on each case. This enhances data comparison and draws the attention to similarities and differences within a case (38).

## Economic Analysis

The economic evaluation will take a pragmatic societal perspective. A matrix, mixed-methods approach will include input from children, young people, staff and other experts. Economic outcomes for the young person are: health-related quality of life defined as a state of complete physical, mental and social well-being, and not merely the absence of disease or infirmity (39); and other indicators of life chances agreed in the evaluability period.

A model will be developed that demonstrates the impact on health and well-being due to the new services/culture compared to before/late implementation using cost utility analysis. A partial economic model will be developed to compare part of the pathway within the Community F:CAMHS model using appropriate structure, process, provider measures, and/or clinical outcome data collected in the qualitative component. A rapid review of costs and consequences, taking a pragmatic societal approach, outside of direct services and in relation to prioritised outcomes, will also be conducted. The Expert Panel will review how representative the data are of lived experience of young people, cares and staff for the purposes of economic analysis. Collaborative considered value judgments will be made about the application of the findings to everyday life in the services. A second systematic review will be carried out to find published utility scores for this population that can be used in the model.

## Ethical Considerations

Ethical approval has been granted by the Health Research Association for the data collected on and from young people or parents/guardians (IRAS project ID: 242383; REC reference: 18/LO/1569). Data collected from staff and referrers has received ethical approval from UCL Ethics (ID: 6087/007).

Digital participant data will be transferred and stored securely on the UCL Data Safe Haven, a secure data platform. Paper copies will be returned and stored securely in locked storage on-site. Access to participants' personal data will be limited to the research team. All data files will be assigned a unique participant identifier to ensure anonymity and stored separately to consent forms. No individual participant will be identified in any presentation or publication. Password-protected, encrypted audio recorders will be used to conduct the interviews, which will be anonymised at point of transcription.

## Discussion and Dissemination

This paper describes the protocol for the national Realist Evaluation of Community F:CAMHS. The primary aim of the present research is to explore whether children and young people accessing early implementer Community F:CAMHS have better mental health outcomes than those accessing late implementer Community F:CAMHS. The secondary aims are to examine the characteristics and outcomes of young people accessing Community F:CAMHS, and explore the experiences of young people, parents/guardians and staff working with or in Community F:CAMHS. Our final aim is to examine the cost effectiveness of the new service model.

The results will be disseminated via internal reports and to the funder (NHS England and NHS Improvement), feedback to participating sites, and more widely through peer-reviewed journal publications and conference presentations (regional and international).

Anticipated limitations of the proposed research include high levels of bias and attrition due to the recruitment techniques (for example, snowball sampling) and possible disengagement with the services or research. Furthermore, the duration of the study is limited and long-term outcomes may not be captured within the study timeframe. Notwithstanding the limitations, the present research is the first of its kind to create a national dataset on children and young people referred to Community F:CAMHS. It is anticipated the findings will contribute to commissioning decisions nationally and will add to the evidence base on the characteristics and needs of children and young people accessing specialist forensic services.

## Data Availability Statement

The original contributions presented in the study are included in the article/supplementary material, further inquiries can be directed to the corresponding author/s.

## Ethics Statement

Ethical approval has been granted by the Health Research Association for the data collected on and from young people or parents/guardians (IRAS project ID: 242383; REC reference: 18/LO/1569). Data collected from staff and referrers has received ethical approval from UCL Ethics (ID: 6087/007). Written informed consent to participate in this study was provided by the participants' legal guardian/next of kin.

## Author Contributions

The protocol was conceived and developed by RL, SD'S, ML, JJ, WR, RU, AR, RS, JW, PFu, DB, JD, DL, PFo, NH, OW, and J-EC. The manuscript was drafted by RL, SD'S, ML, and WR, reviewed and edited by JJ and JE-C, and reviewed by RU, AR, RS, JW, DB, JD, PFu, PFo, DL, NH, and OW. Revisions to the manuscript were made by JJ, RL, SD'S, and JE-C. All authors contributed to the article and approved the submitted version.

## Funding

This work was supported by NHS England and NHS Improvement (grant number not applicable) and sponsored by UCL.

## Conflict of Interest

DL was employed by MindMonkey Associates. WR and RU were employed by company Riches and Ullman LLP. The remaining authors declare that the research was conducted in the absence of any commercial or financial relationships that could be construed as a potential conflict of interest.

## Publisher's Note

All claims expressed in this article are solely those of the authors and do not necessarily represent those of their affiliated organizations, or those of the publisher, the editors and the reviewers. Any product that may be evaluated in this article, or claim that may be made by its manufacturer, is not guaranteed or endorsed by the publisher.
